# Comparative Effects of Calcium, Boron, and Zinc Inhibiting Physiological Disorders, Improving Yield and Quality of *Solanum lycopersicum*

**DOI:** 10.3390/biology13100766

**Published:** 2024-09-26

**Authors:** Bibi Haleema, Syed Tanveer Shah, Abdul Basit, Wafaa M. Hikal, Muhammad Arif, Waleed Khan, Hussein A. H. Said-Al Ahl, Mudau Fhatuwani

**Affiliations:** 1 Floriculture Section, Agriculture Research Institute (ARI), Tarnab 25120, Pakistan; haleema_12@yahoo.co.in; 2 Department of Agriculture, Hazara University, Mansehra 21300, Pakistan; dr.syedtanveershah@hu.edu.pk; 3 Floricultural Biotechnology Laboratory, Department of Horticultural Science, Kyungpook National University, Daegu 41566, Republic of Korea; abdulbasit97_lily@knu.ac.kr; 4 Department of Biology, Faculty of Science, University of Tabuk, Tabuk 71491, Saudi Arabia; wafaahikal@gmail.com; 5 Department of Agronomy, The University of Agriculture, Peshawar 25120, Pakistan; marifkhan75@aup.edu.pk; 6 Laboratory of Crop Production, Department of Applied Biosciences, Kyungpook National University, Daegu 41566, Republic of Korea; waleedkhan.my@gmail.com; 7 Medicinal and Aromatic Plants Research Department, Pharmaceutical and Drug Industries Research Institute, National Research Centre (NRC), 33 El-Behouth St. Dokki, Giza 12622, Egypt; 8 School of Agricultural, Earth and Environmental Sciences, University of KwaZulu-Natal, Private Bag X01, Scottsville, Pietermaritzburg 3209, South Africa

**Keywords:** deficiency, physiological disorders, micronutrients, tomato, correlation

## Abstract

**Simple Summary:**

The tomato is the 2nd major vegetable crop consumed after the potato. It is constantly grown throughout the world, and there is an increasing global demand for it in food industries. Moreover, tomatoes are nutritious vegetables and help to reduce the attack of cancer and cardiovascular diseases. In addition, it remains an economic crop and an important source of income for farmers. Hence, there is a need to meet all the requirements of plant cultivation to obtain greater productivity with good-quality fruits, such as preventing physiological diseases, i.e., blossom end rot and fruit cracking disorders, which reduce marketable tomato yield. More attention should be paid to the factors affecting production to ensure the highest productivity and highest quality. Improving plant nutrition such as calcium, boron, and zinc is one reliable strategy to increase productivity and quality.

**Abstract:**

Localized calcium deficiency at the tomato flower end causes a physiological disorder called blossom end rot, resulting in yield losses of up to 50 percent. Fruit cracking is another physiological disorder of tomatoes that most often occurs when the movement of water and solutes to the tomato is protracted or rapid, but the underlying cause of fruit cracking is, again, calcium deficiency. Therefore, the present field experiment was conducted with the aim of increasing yield and reducing physiological disorders in tomatoes with a foliar application of calcium and micronutrients (zinc and boron). Four levels of calcium (0, 0.3, 0.6, and 0.9%), three levels of boron (0, 0.25, and 0.5%), and three levels of Zinc (0, 0.25, and 0.5%) were applied foliarly three times (starting at flowering, the 2nd application was repeated when the fruits set, and the 3rd after a period of 15 days from the fruits set). An addition of 0.6% calcium increased yield and associated traits with a decreased flower drop. Likewise, a 0.9% calcium addition increased fruit Ca content and decreased blossom end rot, fruit cracking, and Zn content. Foliar spraying with 0.25% boron (compound B) improved flowering and production while reducing flower drop and tomato fruit cracking. Similarly, an application of 0.5% B significantly increased Ca and B content with minimal blossom end rot and Zn content. Likewise, a 0.5% Zn application resulted in yield and yield-related traits with increased fruit B and Zn contents while blossom end rot, fruit cracking, and fruit Ca content were lower when 0.5% of foliar Zn was applied. Therefore, it is concluded that a foliar application of Ca, B, and Zn can be used alone or in combination to minimize the physiological disorders, increase production, and improve tomato fruit quality.

## 1. Introduction

The tomato (*Solanum lycopersicum*) belongs to the Solanaceae family, being the second most consumed vegetable in the world after potatoes [1,2]. It is an economically important plant crop and is grown throughout the world [1]. Tomato cultivation is constantly increasing as a result of the increasing global demand for it and the large number of food industries that are based on it [3]. Tomato production extends from tropical to temperate regions [2]. Global tomato production reached 186.11 million tons in 2022, and Pakistan ranked 26th globally and produced 792,938 tons in the same year [4]. The tomato is a good source of protein, vitamins, minerals, antioxidants, and carotenoids that help in delaying cancer and cardiovascular diseases [3,5].

There are various fungal diseases that attack the tomato such as early and late blight, fusarium wilt, fruit rot, and many others [6], greatly affecting yield and fruit quality of tomatoes. Copper and sulfur-based fungicides are used to control these diseases [7]. Moreover, physiological disorders, especially blossom end rot and fruit cracking, greatly affect tomato productivity and fruit quality [8,9].

Blossom end rot (BER) and fruit cracking disorders decrease marketable tomato yield [8,9]. BER is one of the most devastating physiological disorders affecting various crops, including tomatoes. These physiological disorders can lead to significant yield losses [10]. Tissues affected by BER turn dark brown and become foamy in texture, which may serve as a route for a secondary pathogen [11]. Extensive research into the physiological aspects of the disorder has demonstrated that the underlying causes of BER are related to perturbed calcium (Ca^2+^) homeostasis in the blossom end of the fruit and irregular irrigation conditions in cultivated accessions [10]. Furthermore, abiotic stresses are critical factors in the development of BER, which, combined with unbalanced Ca^2+^ concentrations, significantly influence the severity of the disorder [10].

Fruit cracking is another physiological disorder that occurs in tomatoes when faced with an unsuitable environment, leading to calcium deficiency, which is the underlying cause of fruit cracking [12]. The use of balanced water, fertilizers, and external hormones in production can alleviate cracking to some extent, but it is difficult to fundamentally solve the problem [13,14,15]. Cracking may affect the appearance and quality of the fruits and reduce their shelf life. Cracking fruits are also susceptible to infection with disease-causing bacteria. This severely hampers the production and marketing of tomatoes. Calcium deficiency has been identified as a prominent variable in the risk of various fruit cracking [12]. High Ca in the pericarp cell wall indicates a high concentration of galacturonic acid residues and negatively charged particles, acts as a strengthening agent in the cell wall, and increases the resistance of fruit cracking [12].

More attention must be paid to the factors affecting production to ensure the highest productivity and highest quality. These include plant growth-promoting bacteria (PGPB) [16], cultural practices, field sanitation, phytochemicals [17], and the use of macro and micronutrients [1]. Plant growth-promoting bacteria indirectly helps plants to fix atmospheric nitrogen and better utilizes potassium and phosphates [16]. In this way, they function as growth regulators and stimulate better root development, better seed germination, and higher yield [18]. A plant nutrition plan is considered one of the strategies that can be relied upon to increase productivity. Apart from this, macro- and micro-elements such as calcium, boron, and zinc also play a direct role in the growth and quality of the plant [1,5].

Calcium (Ca) is an important macronutrient that plays structural and physiological roles in plant metabolism [19], regulating many enzymes as a cofactor [20] and assisting in plant growth and development, and enhancing resistance to many abiotic stresses [21]. Calcium deficiency results in the production of less rigid cell walls, which affects homeostasis and stimulates the transduction of chemical signals that cause tissue necrosis [22], thus reducing post-harvest fruit quality and market value. Many physiological disorders in apple, watermelon, pepper, and tomatoes are often attributed to Ca deficiency [23,24].

Boron (B) is one of the essential micronutrients required for good quality and high crop yields, and it is involved in cell wall synthesis and integrity, cell wall fortification, RNA, carbohydrate, phenolic and indole acetic acid, respiration and cell membrane integrity, flowering, and fruit setting [25]. Boron content also affects calcium metabolism and its deficiency reduces calcium bound to pectin constituents [26]. A boron deficiency negatively affects the quality and productivity of many vegetables, including tomatoes [27]. Boron and calcium also stabilize the cell wall structure and are therefore effective in controlling blossom end rot [1,28].

Zinc (Zn) is another important essential micronutrient that plays vital roles in various physiological processes and is crucial for optimal plant growth and development [29]. Zinc is an essential micronutrient required for enzyme activation, protein synthesis, and overall plant growth [30]. It is involved in various metabolic processes, including chlorophyll production, photosynthesis, and carbohydrate metabolism [31].

Keeping in view the importance of Ca, B, and Zn in improving tomato productivity, quality, and reducing the incidence of physiological disorders, the current study was conducted with the following objectives: to evaluate the effect of foliar applied Ca, B, and Zn for improved yield of the tomato; to quantify the incidence of BER and fruit cracking through Ca, B, and Zn management; and to elucidate the interactive effect of Ca, B, and Zn for the maximum decrease in BER and fruit-cracking incidences and improved fruit quality. Foliar spray was selected as a suitable strategy to supply nutrients to plants. It is one of the most important methods because the consumption of nutrients for plants becomes easy and quick by penetrating the stoma and entering in the cells.

## 2. Materials and Methods

The effect of foliar application of calcium (Ca), boron (B), and zinc (Zn) on the growth, yield, and incidence of physiological disorders and the fruit quality of tomatoes was investigated at Agriculture Research Institute (ARI) Tarnab, Peshawar. The experiment was conducted in randomized complete block design (RCBD) with three replications. The experiment consisted of different concentrations of Calcium (0, 0.3, 0.6, and 0.9%), Boron (0, 0.25, and 0.5%), and Zinc (0, 0.25, and 0.5%) applied as a foliar spray three times during the season. The 1st foliar application was performed before the beginning of flowering, the 2nd at fruit set, and the 3rd after 15 days of the fruit setting.

The sources of Ca, B, and Zn used were CaCl_2_.2H_2_O, boric acid, and zinc sulfate, respectively. AnalaR chemicals were used to prepare the nutrient solution. A hand sprayer was used to spray nutrients uniformly onto each plant. Tween-Twenty, a surfactant, was added to the solution at a rate of 0.5 cc/100 mL of water to improve chemical retention. The plants of the control group were sprayed with plain water. All foliar applications were made early in the morning to optimize absorption and long-lasting effects. The seeds of the Riogrande tomato variety were obtained from the National Agriculture Research Council (NARC), Islamabad and nursery reared at ARI Tarnab during the third week of January. The seedlings were hardened and transplanted in the first week of March on one side of the raised bed maintaining a row-to-row distance of 70 cm and a plant-to-plant distance of 30 cm. The plot area was 6.1 m^−2^. The experimental area was fully prepared, and all routine cultural practices, such as weeding and hoeing during crop growth and development, were kept constant and uniform depending on the weather conditions. The nursery plants were grown on raised beds 3 m long and 1 m wide.

The data on the following reproductive, yield, and physiological disorders were recorded.

The flower drop was calculated in percentage. The following formula was used to calculate the parameter.

### 2.1. Reproductive Parameters

#### 2.1.1. Number of Flower Cluster^−1^

The number of flower cluster^−1^ was calculated by the following formula:

$$Number\;of\;flowers\;per\;cluster=\frac{total\;number\;of\;flowers\;from\;tagged\;plants}{total\;number\;of\;flowers\;cluster\;from\;tagged\;plants\;}$$

#### 2.1.2. Number of Fruit Cluster^−1^

The Number of Fruit Cluster^−1^ Was Calculated as Below

$$Number\;of\;fruits\;per\;cluster=\frac{total\;number\;of\;fruits\;from\;tagged\;plants}{total\;number\;of\;fruits\;cluster\;from\;tagged\;plants\;}$$

#### 2.1.3. Number of Flower Clusters Plant^−1^

The number of flower clusters plant^−1^ was noted periodically for the already randomly tagged five plants. After every reading, a colored thread was tied with a stem as a mark for starting the next reading. At the end, the total number of flower clusters was recorded.

#### 2.1.4. Flower Drop (%)

The flower drop was calculated in percentage. The following formula was used to calculate the parameter.

$$Flower\;drop\%=\frac{No.of\;flowers\;per\;cluster-No.of\;fruits\;per\;cluster}{Total\;number\;of\;fruit\;clusters\;per\;plant\;}\times \;100$$

#### 2.1.5. Total Yield (tonnes ha^−1^)

The total yield was calculated in kilograms by weighing all the picked fruits from the tagged plants of plot at random, and yield was then converted to yield in t ha^−1^.

### 2.2. Physiological Disorders

The BER incidence was visually observed at each harvest and calculated as the percentage of total fruits with BER symptoms. The incidence of fruit cracking was visually observed at each harvest and calculated as the percentage of total fruits with signs of fruit cracking.

### 2.3. Nutrient Content of the Fruit

#### 2.3.1. Fruit Calcium Content (%)

To determine the calcium content of fruits, fruits were cut, local tissue was removed and washed again with distilled water. Fruit samples were weighed, dried in the oven at 70 °C, and weighed periodically until the weight became constant. After oven drying, fruit samples were ground using a Tema grinder that was thoroughly cleaned with a brush and acetone for each treatment, and the ground fruit materials were dried with dry ash. Digestion was performing by adding 10 mL of concentrated HNO_3_ and leaving it overnight. Samples were carefully heated on a hot plate until red NO_2_ fumes were no longer produced. The beaker was cooled and a small amount (2–4 mL) of 70% HClO_4_ was added, heated again, and allowed to evaporate to a small volume. The sample was then transferred to a 50 mL flask and diluted to volume with distilled water. The calcium content in leaf samples was measured by the method described by Adrian and Stevens [32] by using an Atomic Absorption Spectrophotometer using model GBC AA 932. The spectrophotometer was calibrated with a standard solution of 5 µg mL^−1^ as per instructions of the manufacturer.

#### 2.3.2. Fruit Boron Content (mg 100 g^−1^ DW)

The fruit boron content was preserved by taking fresh fruit pieces and regenerating the fine tissues. The sample was washed again with distilled water and the initial weight was recorded. The tissue was transferred to an oven heated to 70 °C and weighed periodically to achieve a constant weight. After oven drying, the fruit samples were ground using a Tema dry mill that was cleaned well with a brush and acetone for each treatment and the ground fruit materials were ground with dry ash.

The dry sample (0.5 g) of the plant tissue was placed in a crucible and kept in an oven at a temperature below 450 °C for 5 h to obtain white ash of the sample. Pour 5 mL of 0.5 N HCl in the crucible to mix it well with the dried sample. Filter this sample mixture using butter filter paper. Dilute it to a final volume of 50 mL with 50 mL of distilled water. This solution was used to determine the boron content of fruit by the azomethine-H method [33] using a Shimadzu double beam UV-VIS spectrophotometer (UV-2101 PC, Shimadzu manufacturer, Markham, ON, Canada).

#### 2.3.3. Fruit Zinc Content (mg 100 g^−1^ DW)

The zinc content of fruits was determined by slicing the fruits, removing the local tissues, and then washing them with distilled water. Tissues were weighed and then allowed to dry in the oven at 70 °C until a constant weight was achieved. After oven drying the tissues, the fruit tissues were ground using a Tema mill which was cleaned thoroughly with a brush and acetone for each treatment and ground fruit materials were dried with ash. A total of 1 g of ground dried fruit sample was taken and placed in a small beaker. The digestion was carried out by adding 10 mL of concentrated HNO_3_ and allowed to stand overnight. The samples were heated carefully on a hot plate until the production of red NO_2_ fumes ceased. The beaker was cooled and a small amount (2–4 mL) of 70% HClO_4_ was added to the sample, heated again, and allowed to evaporate to a small volume. Then, the sample was transferred to a 50 mL flask and diluted to volume with distilled water. The zinc content of fruit samples was measured by the procedure described by Adrian and Stevens [32] by using an Atomic Absorption Spectrophotometer using model GBC AA 932. The spectrophotometer was calibrated with a standard solution of 5 µg-mL^−1^ as per instructions of the manufacturer.

### 2.4. Quality or Biochemical Attributes

The fruit firmness was estimated with a Penetrometer (FTFT011, Italian Equipped with a 4 mm probe). Five fruits were taken from each treatment plot at random for this purpose. Smooth and uniform pressure was applied for penetrating the probe into the flesh of the fruit at three regions of tomato: stem end, equatorial region, and blossom end, and then the average was computed. The data were recorded in kg cm^−2^.

The total soluble solids (TSS) were measured at room temperature. A drop of the juice sample was put on the prism of refractometer (Atgo Master-α), and the reading was noted using the unit in “°brix”.

The acidity (%), ascorbic acid (mg 100 g^−1^), reducing and non-reducing sugars (%) were analyzed using the methods reported by Iqtidar and Saleemullah [34].

### 2.5. Statistical Analysis

The data were analyzed statistically using a procedure appropriate for randomized complete block design (RCBD) with split plot arrangement using statistical software Statistix 8 [35]. The means were compared using LSD when the F test was found significant [36]. Moreover, a Pearson correlation was also applied to check the correlation among the studied attributes, following a procedure conducted by Schober et al. [37]. A principal component analysis was also performed using a procedure conducted by Greenacre et al. [38].

## 3. Results

### 3.1. Number of Flowers Cluster^−1^

The foliar application of Ca, B, and Zn significantly affected the number of flowers cluster^−1^ of the tomato. The Ca × B and B × Zn interactions were significant while Ca × Zn and Ca × B × Zn interactions were not significant for the number of flowers cluster^−1^. The number of flowers in cluster^−1^ significantly (*p* ≤ 0.05) increased from 4.82 to 6.33 with 0% to 0.6% Ca as foliar spray but increased the Ca concentration to 0.9% significantly (*p* ≤ 0.05) and reduced the number of cluster^−1^ flowers (5.37). The number of cluster^−1^ flowers significantly (*p* ≤ 0.05) increased from 4.97 to 6.05 with the increase in B concentration from 0 to 0.25%. However, increasing B concentration to 0.5% reduced the number of flowers cluster^−1^ (5.50). The number of flowers cluster^−1^ of tomato also increased with the increasing concentration of Zn in the foliar solution. The flowers cluster^−1^ increased from 5.03 to 6.06 with the increasing Zn concentration from 0 to 0.5%, followed by flowers cluster^−1^ (5.44) with a foliar application of 0.25% Zn solution (Table 1).

**Table 1:** Effect of calcium, boron, and zinc on flowers cluster^−1^, fruit cluster^−1^, and flower clusters plant^−1^ of tomato.

Calcium Levels (%)	No. of Flowers Cluster^−1^	No. of Fruit Cluster^−1^	No. of Flower Cluster Plant^−1^
0	4.82 c	3.74 c	13.19 c
0.3	5.52 b	4.29 b	14.82 b
0.6	6.33 a	4.82 a	15.07 b
0.9	5.37 b	4.19 b	16.78 a
LSD at α 0.05	0.45	0.33	0.68
Boron (%)			
0	4.97 c	3.75 c	12.19 c
0.25	6.05 a	4.97 a	15.28 b
0.5	5.50 b	4.06 b	17.42 a
LSD at α 0.05	0.39	0.29	0.59
Zinc (%)			
0	5.03 c	4.00 b	13.86 b
0.25	5.44 b	4.14 b	14.39 b
0.5	6.06 a	4.65 a	16.64 a
LSD at α 0.05	0.39	0.29	0.59
Interactions			
Ca × B	Figure 1a	Figure 1b	Figure 2
Level of Significance	**	**	**
Ca × Zn	---	---	Figure 2
Level of Significance	Ns	NS	*
B × Zn	Figure 1a	Figure 1b	Figure 2
Level of Significance	*	**	**
Ca × B × Zn	---	---	---
Level of Significance	NS	NS	NS

Means followed by similar letter(s) in column do not differ significantly from one another. NS = non-significant and *, ** = Significant at 5, and 1% level of probability, respectively.

**Figure 1:**
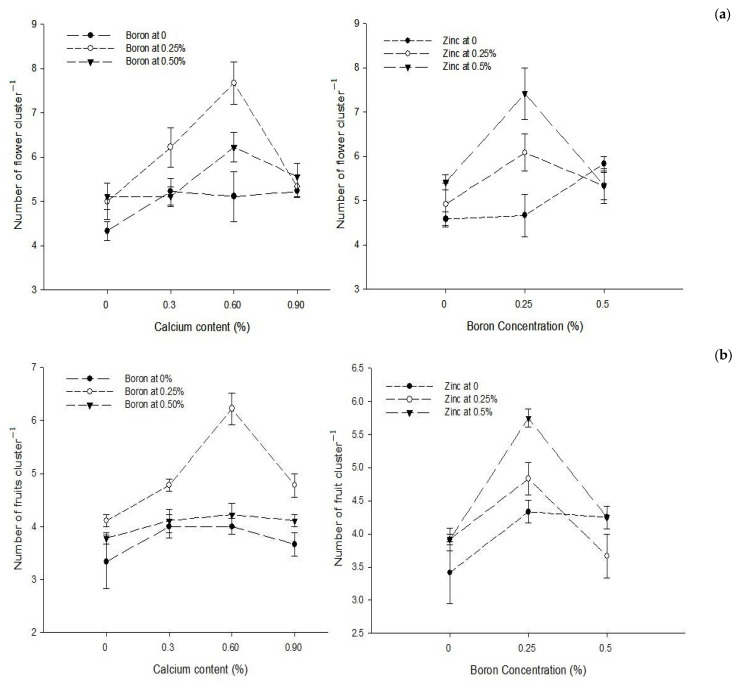
The interactions of zinc, boron, and calcium on (**a**) number of flowers cluster^−1^ and (**b**) number of fruits cluster^−1^ of tomato. The vertical bars represent standard error.

**Figure 2:**
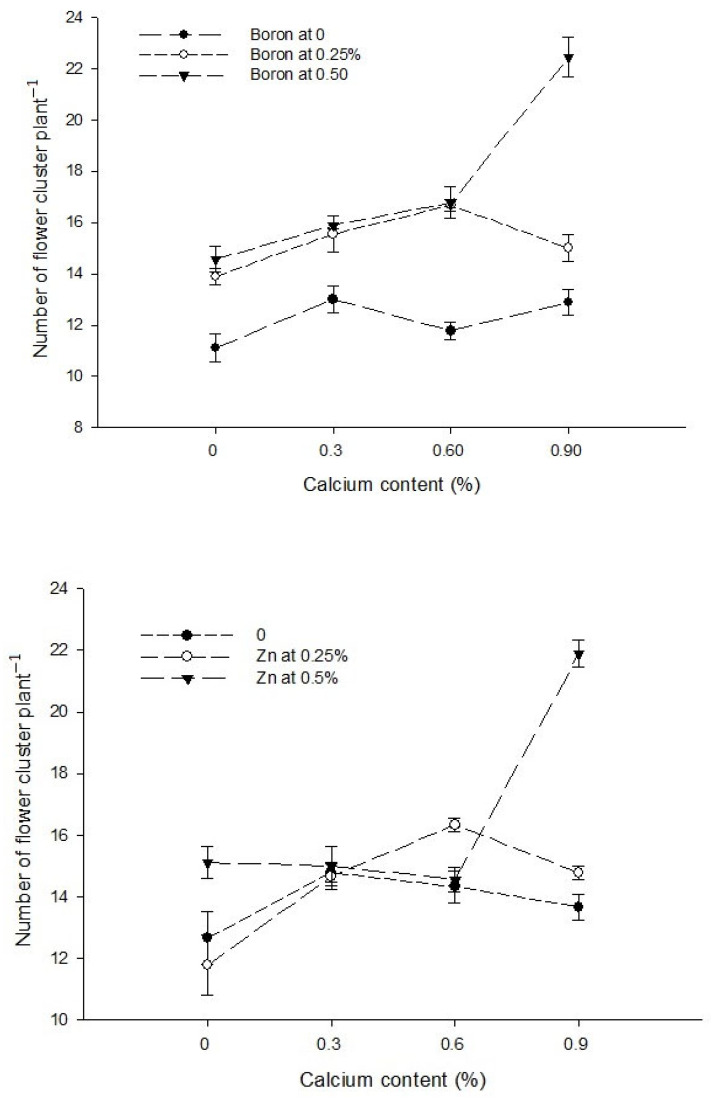

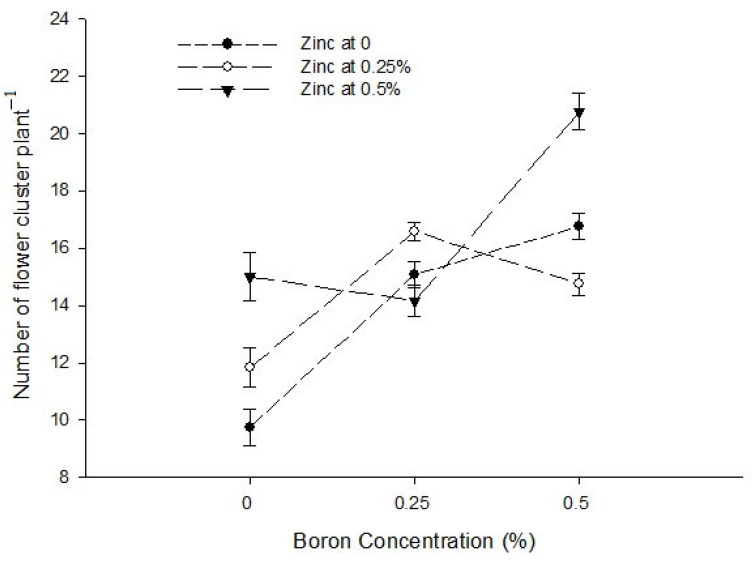
The interactions of zinc, boron, and calcium on number of flowers cluster plant^−1^ of tomato. The vertical bars represent standard error.

The interaction between Ca and B indicated that number of flowers cluster^−1^ of tomato was significantly (*p* ≤ 0.05) higher (7.66) with the combined application of 0.25% B and 0.6% Ca. The interaction between B and Zn indicated that number of flowers cluster^−1^ of tomato was significantly (*p* ≤ 0.05) higher (7.42) with a foliar application of 0.25% B + 0.5% Zn (Figure 1a).

### 3.2. Number of Fruit Cluster^−1^

The statistical analysis of the data revealed that a foliar application of Ca, B, and Zn significantly (*p* ≤ 0.05) affected the number of fruit cluster^−1^. The Ca × B and B × Zn interactions were also significant (*p* ≤ 0.05), while Ca × Zn and Ca × B × Zn interactions were not significant for the number of fruits cluster^−1^. The number of fruits cluster^−1^ significantly (*p* ≤ 0.05) increased from 3.74 to 4.82 with the increasing Ca concentration from 0 to 0.6%, but a further increase in the Ca concentration to 0.9% decreased the number of fruits cluster^−1^ (6). The number of fruits cluster^−1^ of tomato significantly (*p* ≤ 0.05) increased from 3.75 in control plants that increased to the maximum of 4.97 with the application of 0.25% B as foliar spray but declined to 4.06 with an increase in foliar B concentration to 0.5%. The number of fruit cluster^−1^ of tomato continually increased (*p* ≤ 0.05) with the increasing concentration of Zn from 0 to 0.5%. The maximum number of fruits cluster^−1^ 4.65 was recorded at the highest Zn concentration of 0.5% and the least number of fruits cluster^−1^ (4.0) were recorded in control plants that increased to 4.14 and 4.50 with 0.25 and 0.5% Zn applied as foliar spray (Table 1).

The interaction between Ca and B was found significant (*p* ≤ 0.05). Plants treated with 0.6% Ca and 0.25% B resulted in the maximum number of fruits cluster^−1^ (6.22) compared to the control (3.33) fruits cluster^−1^. The interaction between B and Zn was also found to be significant (*p* ≤ 0.05) and indicated that fruits cluster^−1^ increased from 3.42 in control to 5.75 in plants sprayed with 0.25% B + 0.5% Zn (Figure 1b).

### 3.3. Number of Flower Clusters Plant^−1^

The data in relation to the number of flower clusters plant^−1^ are presented in Table 1. The data analysis indicated that a foliar application of Ca, B, and Zn significantly affected the number of flower clusters plant^−1^ of tomato. The Ca × B, Ca × Zn, and B × Zn interactions were significant, while the Ca × B × Zn interaction was not significant for the number of flower clusters plant^−1^. The number of flower clusters plant^−1^ increased from 13.19 in the control group to 16.78 in plants sprayed with a 0.9% calcium solution. A statistically similar number of plant flower clusters ^−1^ observed with a foliar application of 0.3 and 0.6% Ca solutions were 14.82 and 15.07, respectively. Similarly, the number of flower clusters plant^−1^ of tomato increased with an increasing B concentration in foliar application. The minimum number of flower cluster plant^−1^ (12.19) was counted in control plants which increased to 15.28 and 17.42 in the plants that received a foliar application of 0.25 and 0.5% B, respectively. The number of flower clusters plant^−1^ was 13.86 and 14.39 with a foliar application of 0 and 0.25% Zn solutions, respectively, with the difference being non-significant. However, a further increase in the Zn concentration of foliar spray to 0.5% increased the number of flower clusters plant^−1^ to the maximum of 16.64 (Table 1).

The interaction between Ca and B indicated that the number of flower clusters plant^−1^ was significantly (*p* ≤ 0.05) maximum (22.44) in plants sprayed with 0.9% Ca + 0.5% B (Figure 2). The interaction between Ca and Zn was also found to be significant (*p* ≤ 0.05) and revealed that the number of flower clusters plant^−1^ of tomato was higher (21.89) with a foliar application of 0.5% Zn + 0.9% Ca on plants (Figure 2). The interaction between B and Zn showed that the number of flowers cluster^−1^ was higher when0.5% B was applied at all Zn levels. The flower clusters plant^−1^ was also found significant (*p* ≤ 0.05) and showed that the maximum number of flower clusters plant^−1^ (20.75) was found in plants treated with 0.5% B + 0.5% Zn (Figure 2).

### 3.4. Flower Drop (%)

The data on the flower drop of the tomato plant in relation to different combinations of foliar Ca, B, and Zn application are presented in Table 2. The foliar application of Ca and B significantly affected flower drop of the tomato plant, but the influence of the foliar Zn application was not significant. The Ca × B and B × Zn interactions were significant while the Ca × Zn and Ca × B × Zn interaction was not significant for flower drop of the tomato plant. Flower drop decreased from the highest (33.74%) in control plants (0% Ca) to the minimum (18.85%) when the Ca concentration of the foliar spray was increased to 0.6%. However, a higher Ca concentration (0.9%) increased the flower drop again though it was lower (25.69%) than control. Likewise, flower drop decreased from 31.50% to 17.86% with an increase in B concentration from 0 to 0.25%. However, a further increase in B concentration to 0.5% again increased flower drop to 26.50% (Table 2).

**Table 2:** Effect of calcium, boron, and zinc on percent flower drop, yield, blossom end rot, and fruit cracking in tomato.

Calcium Levels (%)	Flower Drop (%)	Yield (t ha^−1^)	Blossom End Rot (%)	Fruit Cracking (%)
0	33.74 a	19.96 c	16.93 a	6.74 a
0.3	22.85 bc	23.89 b	15.00 b	5.85 b
0.6	18.85 c	28.11 a	11.85 c	5.26 c
0.9	25.69 b	23.04 bc	6.70 d	3.63 d
LSD at α 0.05	4.52	3.76	0.72	0.52
Boron (%)				
0	31.50 a	19.17 c	14.00 a	6.50 a
0.25	17.86 c	28.30 a	12.41 b	4.44 c
0.5	26.50 b	23.78 b	11.44 c	5.17 b
LSD at α 0.05	3.92	3.25	0.623	0.45
Zinc (%)				
0	27.81	19.14 c	15.19 a	5.97 a
0.25	24.33	23.30 b	11.67 b	5.31 b
0.5	23.72	28.80 a	11.00 c	4.83 c
LSD at α 0.05	NS	3.25	0.62	0.45
Interactions				
Ca × B	Figure 3a	Figure 3b	Figure 4	Figure 5
Level of Significance	**	**	**	**
Ca × Zn	---	---	Figure 4	Figure 5
Level of Significance	NS	NS	**	*
B × Zn	Figure 3a	Figure 3b	Figure 4	Figure 5
Level of Significance	**	*	*	**
Ca × B × Zn	---	---	---	---
Level of Significance	NS	NS	NS	NS

Means followed by similar letter(s) in column do not differ significantly from one another. NS = non-significant and *, ** = Significant at 5 and 1% level of probability, respectively.

**Figure 3:**
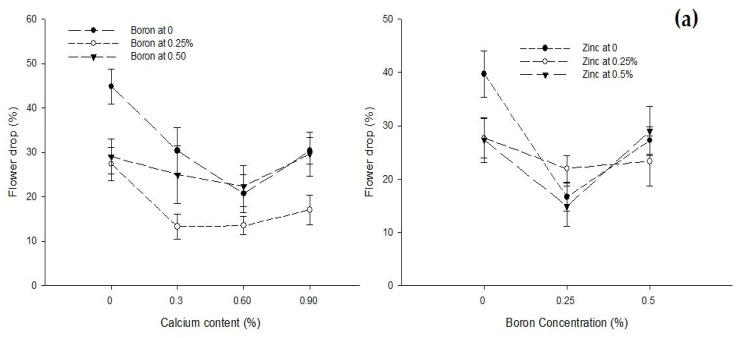

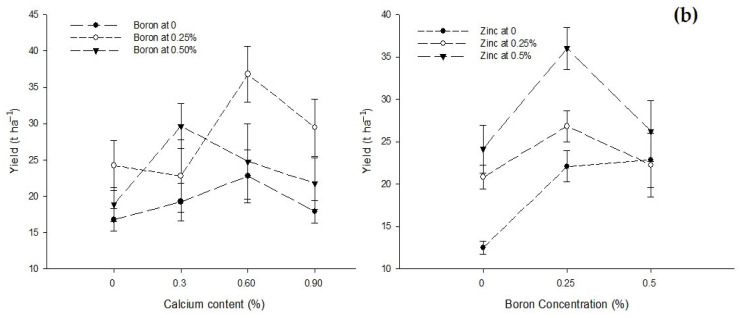
The interaction of Ca, B, and Zn application on the (**a**) percent flower drop and (**b**) yield of tomato. The vertical bars represent standard error.

**Figure 4:**
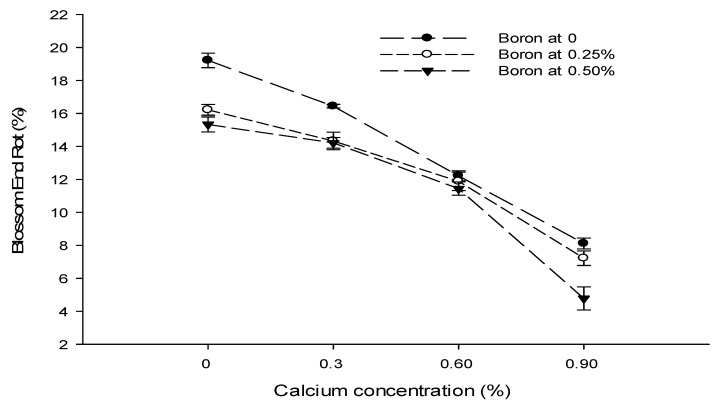

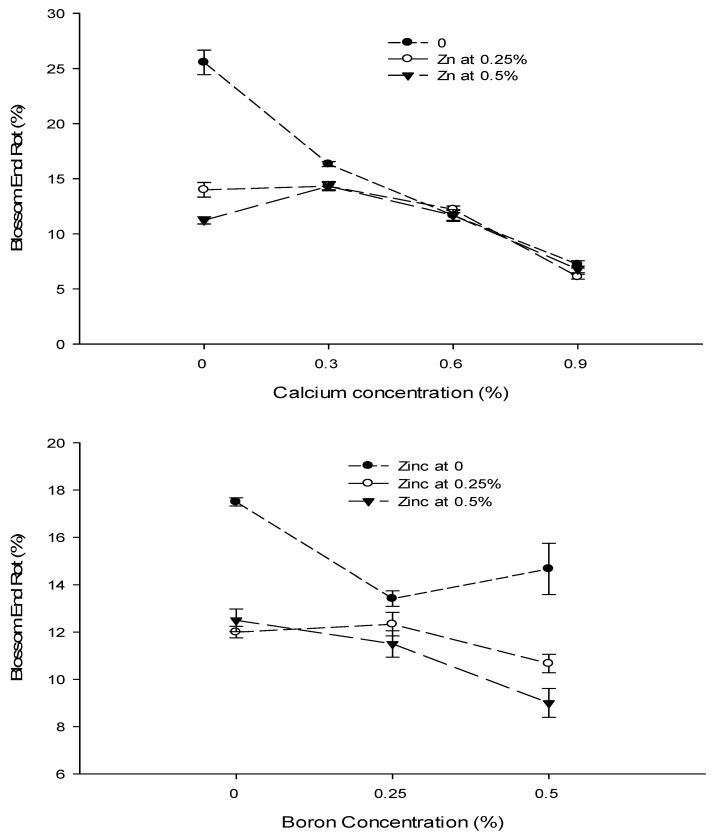
The interaction of Ca, B, and Zn application on blossom end rot of tomato. The vertical bars represent standard error.

**Figure 5:**
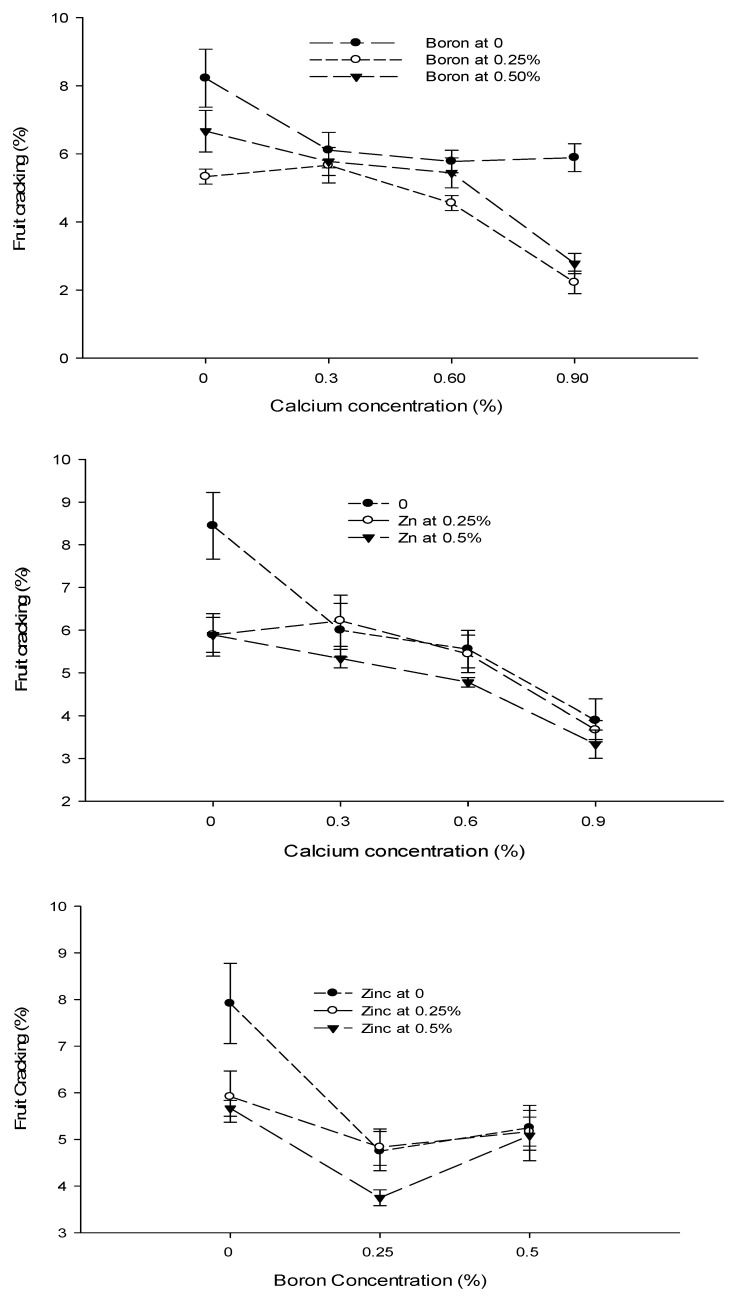
The interaction of Ca, B, and Zn application on fruit cracking of tomato. The vertical bars represent standard error.

The interaction (*p* ≤ 0.05) between Ca and B showed that flower drop significantly (*p* ≤ 0.05) decreased with an increasing concentration of Ca from 0 to 0.3% at all B levels, but a further increase in the Ca concentration increased the flower drop. The minimum flower drop (13.33%) was observed in plants sprayed with 0.3% Ca + 0.25% B. The interaction between B and Zn was also found significant (*p* ≤ 0.05). The flower drop of tomato was lower (14.89%) in plants treated with 0.25% B + 0.25% Zn (Figure 3a).

### 3.5. Yield (t ha^−1^)

Tomato crop production was significantly affected by foliar application with Ca, B, and Zn. The interactions between Ca × B and B × Zn were significant while Ca × Zn and Ca × B × Zn interactions were not significant for tomato yield (Table 2). The yield of the control treatment (0% Ca) was 19.96 t/ha, which increased to 28.11 t ha^−1^ with an application of 0.6% Ca, but a further increase in Ca concentration to 0.9% decreased tomato yield to 23.04 t ha^−1^. The foliar application of B also increased the yield from 19.17 t ha^−1^ in control plants to 28.30 t ha^−1^ h with the foliar application of 0.25% B. However, the higher B concentration (0.5%) decreased the yield to 23.78 t ha^−1^. Tomato production also increased with an increasing Zn concentration. The least yield of 19.14 t ha^−1^ recorded in the control treatment increased to the maximum yield of 28.80 t ha^−1^ with 0.5% Zn application (Table 2).

The interaction between Ca and B showed significant (*p* ≤ 0.05) differences for yield (t ha^−1^) of tomato. The maximum yield (36.78 t ha^−1^) was recorded in plants treated with 0.6% Ca + 0.25% B. The interaction between B and Zn indicated that the yield of tomato was significantly (*p* ≤ 0.05) higher (36.00 t ha^−1^) in plants sprayed with 0.25% B + 0.5% Zn when compared to the least yield of 12.50 t ha^−1^ in control plants (Figure 3b).

### 3.6. Blossom End Rot (BER) (%)

The statistical analysis of the data revealed that a foliar application of Ca, B, and Zn significantly affected the blossom end rot of tomato fruit. The interactions among Ca × B, Ca × Zn, and B × Zn were significant, while the Ca × B × Zn interaction was not significant for blossom end rot. The blossom end rot (BER) incidence decreased significantly with an increase in the Ca concentration from 0 to 0.9%. The lowest incidence of BER of 6.70% was recorded at 0.9% Ca application followed by 11.85% BER incidence in plants sprayed with 0.6% Ca solution. The highest BER incidence (16.93%) was recorded in control plants. The BER incidence of tomato decreased significantly with an increasing B concentration from 0 to 0.5%. The lowest incidence of BER (11.44%) was recorded with a foliar application of 0.5% B, followed by 12.41% with 0.25% B application. The highest BER incidence of 14.00% was recorded in control plants. The BER incidence also decreased with an increasing concentration of Zn from 0 to 0.5%. The lowest incidence of blossom end rot (11.00%) was recorded with the application of 0.5% Zn solution to the plants, followed by 11.67% with a 0.25% Zn application. The highest BER incidence (15.19%) was recorded in control (Table 2).

The interaction between Ca and B was found significant (*p* ≤ 0.05) for BER incidence of tomato. The minimum (4.78%) was observed in plants supplied with a foliar application of 0.9% Ca + 0.5% B. The interaction between Ca and Zn was also found to be significant (*p* ≤ 0.05). The BER incidence of tomato was found to be the minimum (6.11%) in plants treated with a 0.25% Zn + 0.9% Ca concentration (Figure 4). The interaction between B and Zn showed that a 0.5% B concentration resulted in significantly (*p* ≤ 0.05) lower BER incidence (9.1%) in plants treated with a 0.5% B + 0.5% Zn concentration (Figure 4).

### 3.7. Fruit Cracking (%)

The data concerning the fruit cracking are given in Table 2. The analysis of data showed that a foliar application of Ca, B, and Zn significantly affected the cracking of tomato fruit. The interactions among Ca × B, Ca × Zn, and B × Zn were significant, while the Ca × B × Zn interaction was not significant for the fruit cracking of tomato. The fruit cracking of tomato decreased with an increase in Ca concentration from 0 to 0.9%. The highest fruit cracking (6.74%) was recorded in control and declined to 5.26% with the foliar application of 0.6% Ca solution. The least fruit cracking (3.63%) was recorded at a 0.9% Ca application to the plants. The tomato fruit cracking also decreased significantly when increasing the concentration of B from 0 to 0.25% in the foliar spray solution. The lowest fruit cracking of 4.44% was observed with the application of 0.25% B as foliar spray, followed by fruit cracking of 5.17% with a 0.5% B application. The highest fruit cracking incidence (6.50%) was recorded in fruits harvested from control plants. The fruit cracking of tomato also decreased significantly with an increasing concentration of Zn from 0 to 0.5%. The lowest fruit cracking (4.83%) was recorded with the application of 0.5% Zn as foliar spray, followed by 5.31% at a 0.25% Zn application. The highest fruit cracking (5.97%) was recorded in control (Table 2).

The interaction between Ca and B was found significant (*p* ≤ 0.05) for fruit cracking. The lowest fruit cracking (2.22%) was observed in plants that were sprayed with a 0.9% Ca + 0.25% B concentration (Figure 5). The interaction between Ca and Zn indicated significant differences (*p* ≤ 0.05) and reported a decrease in fruit cracking (3.33%) with a foliar application of 0.9% Ca +0.5% Zn. The interaction between B and Zn indicated that fruit cracking of tomato was significantly (*p* ≤ 0.05) decreased (3.75%) in tomato plants treated with a 0.25% B + 0.5% Zn concentration (Figure 5).

### 3.8. Fruit Calcium Content (mg 100 g^−11^ DW)

The analysis of data revealed that a foliar application of Ca, B, and Zn significantly affected the fruit Ca content of tomato. The Ca × B interaction was significant while Ca × Zn, B × Zn, and Ca × B × Zn interactions were not significant for fruit Ca content. The fruit Ca content increased when increasing the Ca concentration of the foliar spray from 0 to 0.9%. The mean fruit Ca content was the minimum (8.66 mg 100 g^−1^ DW) in control. The fruit calcium content increased to 9.43 and 9.94 mg 100 g^−1^ DW when increasing the Ca concentration to 0.3 and 0.6%. The maximum fruit Ca content 10.21 mg 100 g^−1^ DW was recorded at the application of 0.9% Ca with the difference being non-significant between 0.6 and 0.9% calcium solution. The fruit Ca content also increased with an increase in the concentration of B as foliar spray. The least mean Ca content (9.12 mg 100 g^−1^ DW) of control fruits increased to 9.59 and 9.97 mg 100 g^−1^ DW with a foliar application of 0.25 and 0.50% B, respectively (Table 3). The foliar application of Zn decreased the fruit Ca content with an increase in Zn concentration from 0 to 0.5%. The highest fruit Ca content of 8.70 mg 100 g^−1^ DW was recorded in control, which was declined to 7.10 and 6.88 mg 100 g^−1^ DW with a foliar application of 0.25 and 0.5% Zn, respectively, to the tomato plants. The difference in the calcium content of tomato fruit with 0.25 and 0.50% Zn foliar application was, however, non-significant (Table 3).

**Table 3:** Effect of calcium, boron, and zinc on fruit calcium content, fruit boron content, and fruit zinc content of tomato.

Calcium Levels (%)	Fruit Calcium Content (mg 100 g^−1^ DW)	Boron Content (mg 100 g^−1^ DW)	Zinc Content (mg 100 g^−1^ DW)
0	8.66 c	2.83	2.43 a
0.3	9.43 b	2.99	2.34 a
0.6	9.94 a	2.97	2.28 ab
0.9	10.21 a	3.19	2.08 b
LSD at α 0.05	0.316	NS	0.22
Boron (%)			
0	9.12 c	2.64 b	2.41 a
0.25	9.59 b	2.99 a	2.26 ab
0.5	9.97 a	3.24 a	2.18 b
LSD at α 0.05	0.27	0.31	0.19
Zinc (%)			
0	8.70 a	2.78 b	1.81 b
0.25	7.10 b	2.93 b	2.45 a
0.5	6.88 b	3.27 a	2.59 a
LSD at α 0.05	0.27	0.31	0.191
Interactions			
Ca × B	Figure 6a	Figure 6b	Figure 7
Level of Significance	**	*	**
Ca × Zn	---	---	Figure 7
Level of Significance	NS	NS	**
B × Zn	---	---	Figure 7
Level of Significance	NS	NS	*
Ca × B × Zn	---	---	---
Level of Significance	NS	NS	NS

Means followed by similar letter(s) in column do not differ significantly from one another. NS = non-significant and *, ** = Significant at 5 and 1% level of probability, respectively.

**Figure 6:**
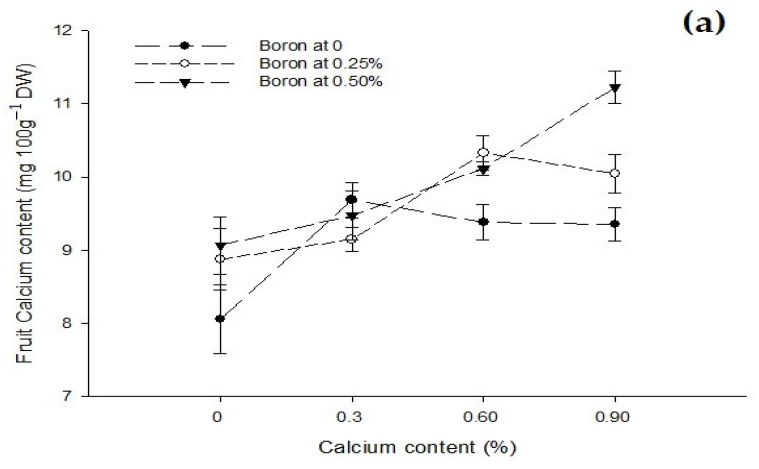

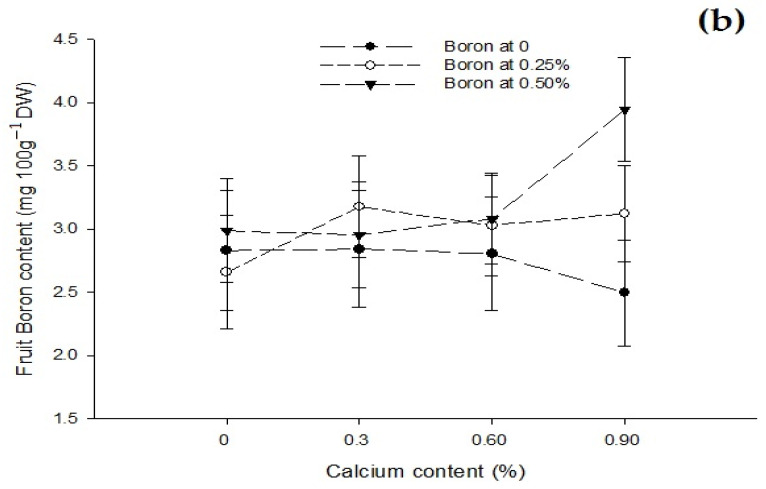
The interaction effects of Ca, B, and Zinc application on calcium (**a**) and boron (**b**) contents of the tomato fruit. The vertical bars represent standard error.

**Figure 7:**
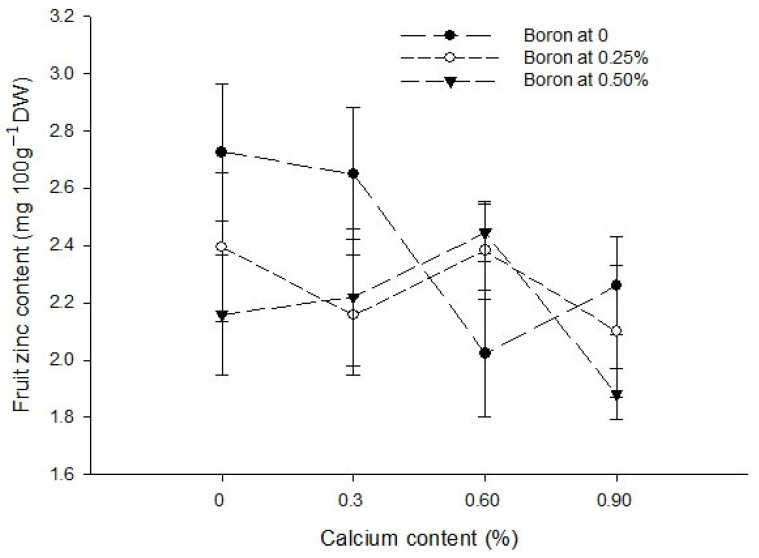

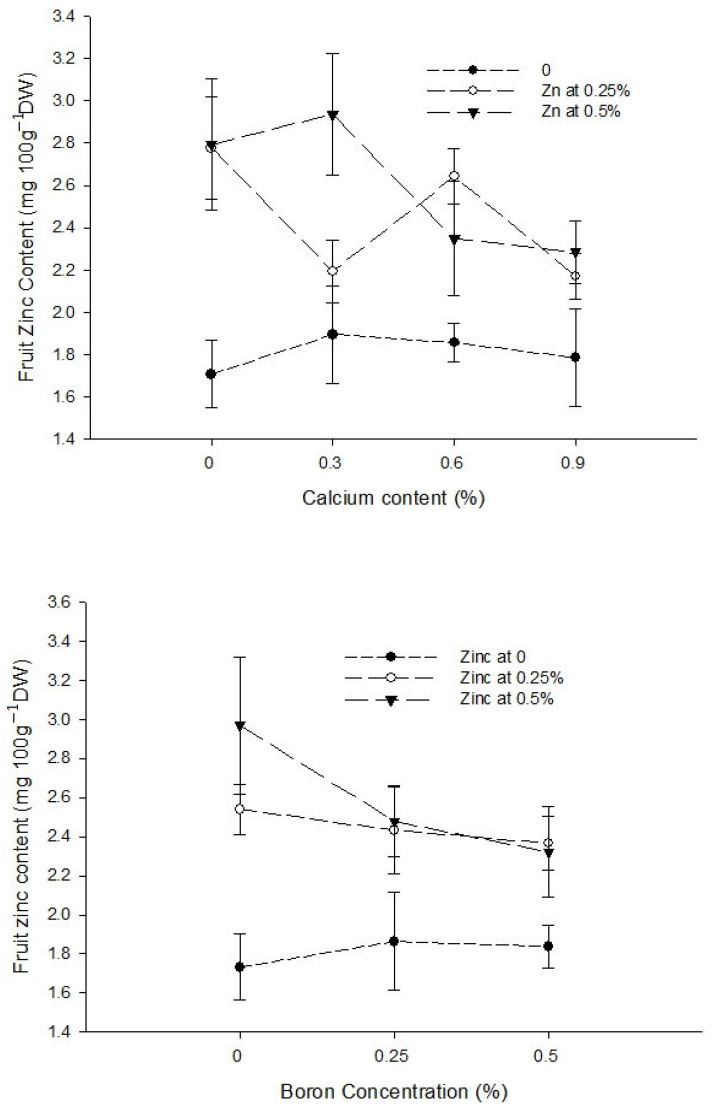
The interaction effects of Ca, boron, and Zn application on the fruit Zinc content of tomato. The vertical bars represent standard error.

The interaction between Ca and B revealed that fruit Ca content was significantly (*p* ≤ 0.05) increased to 11.23 mg 100 g^−1^ DW when increasing the B and Ca concentration to 0.5% and 0.9%, respectively, as compared to control (8.35 mg 100 g^−1^ DW) (Figure 6a).

### 3.9. Fruit Boron Content (mg 100 g^−1^ DW)

The data related to fruit B content are given in Table 3. The statistical analysis of the data revealed that the foliar application of B and Zn significantly affected the B content of tomato fruit. The effect of the Ca foliar application on fruit B content was not significant. The Ca × B interaction was significant while Ca × Zn, B × Zn, and Ca × B × Zn interactions were not significant for fruit boron content. The mean B content of tomato fruit increased with an increase in concentration of B. The lowest B content of 2.64 mg 100 g^−1^ DW was recorded in control fruit that increased to 2.99 mg 100 g^−1^ DW at a 0.25% B application and finally to the highest (3.24 mg 100 g^−1^ DW) recorded with a foliar application of 0.5% (Table 3). The fruit B content increased non-significantly from 2.78 to 2.93 mg 100 g^−1^ with a foliar application of 0 to 0.25% Zn. However, the foliar application of 0.5% Zn increased fruit B content significantly to 3.27 mg 100 g^−1^ DW (Table 3). The interaction between Ca and B showed significant (*p* ≤ 0.05) that B content of tomato fruit was found maximum (3.94 mg 100 g^−1^ DW) in plants that was given 0.9% Ca + 0.5% B as foliar spray (Figure 6b).

### 3.10. Fruit Zinc Content (mg 100 g^−1^ DW)

The data regarding the Zn content of fruit are given in Table 3. The analysis of the data indicated that a foliar application of Ca, B, and Zn significantly affected the fruit Zn content of tomato. The Ca × B, Ca × Zn, and B × Zn interactions were significant, while the Ca × B × Zn interactions was not significant for the fruit Zn content of tomato. The means across Ca treatment revealed that the foliar application of Ca decreased the Zn content of tomato fruit. The highest Zn content (2.42 mg 100 g^−1^ DW) in control plants decreased to 2.34 mg 100 g^−1^ DW with a foliar application of 0.3% Ca. The difference in control and 0.3% Ca treatment was, however, non-significant. The fruit zinc content declined further to 2.28 and 2.08 mg 100 g^−1^ DW with 0.6 and 0.9% Ca applied as a foliar spray to the plants (Table 3). The foliar application of B also decreased the Zn content of tomato fruit, which was the highest (2.41 mg 100 g^−1^ DW) in control and declined to 2.26 and 2.18 mg 100 g^−1^ DW with a foliar application of 0.25 and 0.5% B, respectively. The difference in Zn content with a 0.25 and 0.5% B application was, however, non-significant (Table 3). The foliar application of Zn increased the Zn content of tomato fruit significantly from the minimum 1.81 mg 100 g^−1^ DW in control to 2.45 and 2.59 mg 100 g^−1^ DW in fruit harvested from plants sprayed with 0.25 and 0.5% Zn, respectively. The difference in Zn content of the fruit with 0.25 and 0.5% Zn application was, however, non-significant (Table 3).

The interaction between Ca and B indicated that fruit Zn content significantly (*p* ≤ 0.05) decreased with an increasing concentration of Ca and B concentration. The Zn content of control fruit 2.73 mg 100 g^−1^ DW decreased significantly (*p* ≤ 0.05) to 1.88 mg 100 g^−1^ DW in fruits harvested from plants sprayed with a combination of 0.9% Ca + 0.5% B (Figure 7). The interaction between Ca and Zn revealed that Zn content of tomato fruit significantly (*p* ≤ 0.05) increased with 0.3% Ca and an increasing Zn concentration in the foliar solution. The maximum (2.94 mg 100 g^−1^ DW) in fruits harvested from plants received a combination of 0.3% Ca + 0.5% Zn (Figure 7). The interaction between B and Zn indicated significant (*p* ≤ 0.05) differences for fruit zinc content and found that a combined application of 0.5% Zn and 0.5% B resulted in maximum Zn content (2.32 mg 100 g^−1^ DW) (Figure 7).

### 3.11. Fruit Firmness (kg cm^−2^)

The foliar application of Ca, B, and Zn significantly affected the fruit firmness of tomato. The Ca × B interaction was significant while the Ca × Zn, B × Zn, and Ca × B × Zn interactions were not significant for fruit firmness (Table 4).

The fruit firmness increased with an increase in Ca concentration from 0 to 0.9%. The maximum fruit firmness 2.99 kg cm^−2^ was recorded at 0.9% level of Ca, followed by the statistically same fruit firmness of 2.67 kg cm^−2^ and 2.80 kg cm^−2^ at 0.3 and 0.6% Ca concentrations, respectively. The minimum fruit firmness 2.37 kg cm^−2^ was recorded in control (Table 4). The fruit firmness of tomato was statistically at par (2.57 and 2.70 kg cm^−2^) with 0 to 0.25% B applications but was significantly enhanced to the highest of 2.86 kg cm^−2^ with the foliar application of 0.5% B. (Table 4). The highest fruit firmness of tomato was found in control plants (2.82 kg cm^−2^). However, the fruit firmness of tomato decreased significantly to 2.56 kg cm^−2^ with the application of 0.5% Zn as a foliar solution (Table 4).

**Figure 8:**
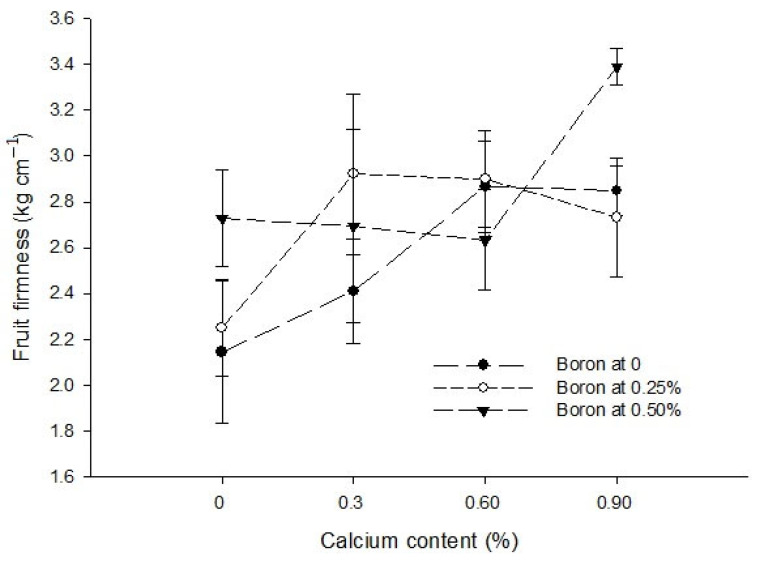
The interaction effects of Ca, boron and Zn application on the fruit firmness of tomato. The vertical bars represent standard error.

The interaction between Ca and B significantly (*p* ≤ 0.05) increased the fruit firmness at all the levels of Ca and B. However, the highest fruit firmness (3.38 kg cm^−2^) was measured at 0.5% B + 0.9% Ca. By contrast, the fruit firmness in control treatment was 2.14 kg cm^−2^ (Figure 8).

**Table 4:** Effect of calcium, boron, and zinc on fruit firmness, total soluble solids, percent acidity, and TSS to acid ratio of tomato.

Calcium Levels (%)	Fruit Firmness (kg cm^−2^)	Total Soluble Solids (°brix)	Percent Acidity (%)	TSS Acid Ratio
0	2.37 c	4.45 a	0.27	18.27
0.3	2.67 b	4.17 b	0.31	15.80
0.6	2.80 ab	3.82 c	0.29	16.84
0.9	2.99 a	3.38 d	0.37	17.29
LSD at α 0.05	0.24	0.24	NS	NS
Boron (%)				
0	2.57 b	4.37 a	0.27	17.95
0.25	2.70 ab	3.93 b	0.32	15.98
0.5	2.86 a	3.56 c	0.34	17.22
LSD at α 0.05	0.21	0.21	NS	NS
Zinc (%)				
0	2.82 a	3.36 c	0.28	16.65
0.25	2.75 ab	3.89 b	0.27	18.28
0.5	2.56 b	4.62 a	0.39	16.22
LSD at α 0.05	0.21	0.21	NS	NS
Interactions				
Ca × B	Figure 8	Figure 9	---	Figure 10
Level of Significance	**	NS	NS	**
Ca × Zn	---	----	---	---
Level of Significance	NS	**	NS	NS
B × Zn	---	Figure 9	---	---
Level of Significance	NS	**	NS	NS
Ca × B × Zn	---	---	---	---
Level of Significance	NS	NS	NS	NS

Means followed by similar letter(s) in column do not differ significantly from one another. NS = non-significant and ** = Significant at 1% level of probability.

**Figure 9:**
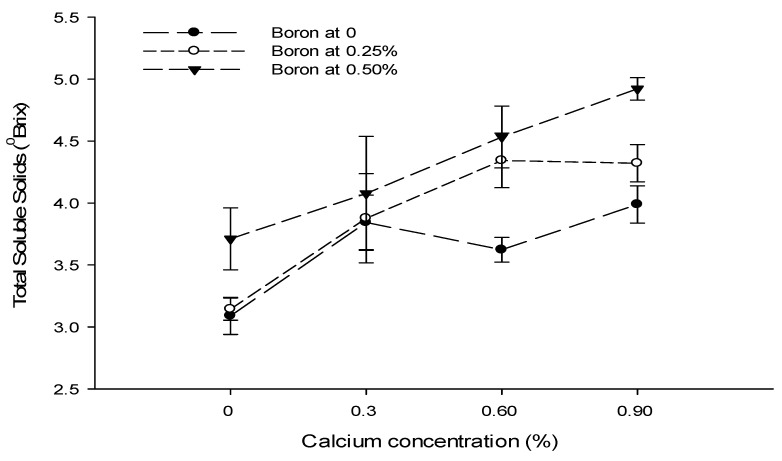

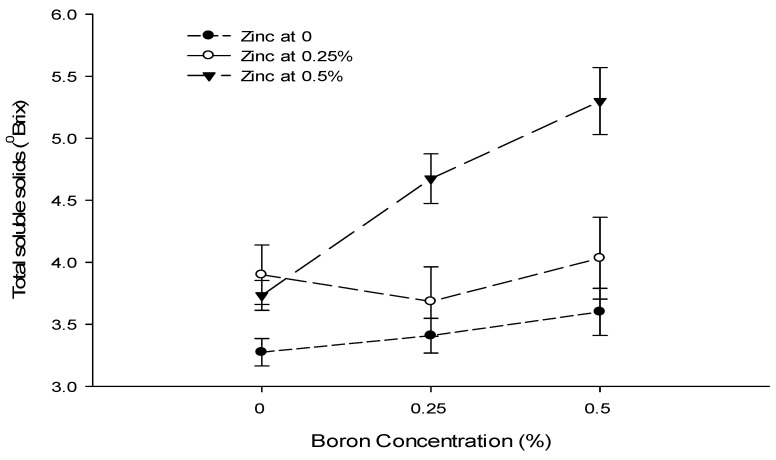
The interaction effects of Ca, boron, and Zn application on the total soluble solids of tomato. The vertical bars represent standard error.

**Figure 10:**
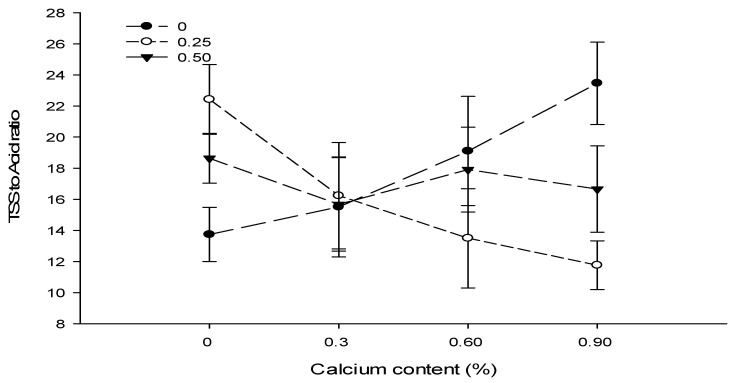
The interaction effects of Ca, boron, and Zn application on the TSS to acid ratio of tomato. The vertical bars represent standard error.

### 3.12. Total Soluble Solids (°brix)

The mean data table indicated that a foliar application of Ca, B, and Zn significantly affected the total soluble solid content of tomato. The Ca × B and B × Zn interactions were significant, while the Ca × Zn and Ca × B × Zn interactions were not significant for the total soluble solid content of tomato (Table 4).

The total soluble solid content of tomato fruit decreased from 4.45 to 3.38 °brix in untreated fruits with 0.9% Ca as a foliar spray (Table 4). The mean TSS content of tomato fruit decreased with an increasing B concentration. The highest TSS content (4.37 °brix) was recorded in control fruits followed by a TSS content of 3.93 °brix with the application of 0.25% B as foliar spray. The lowest TSS content (3.56 °brix) was recorded in fruits sprayed with a 0.5% B concentration (Table 4). The TSS content of tomato gradually increased with an increasing concentration of Zn. The least TSS content of 3.36 °brix was measured in control fruits and increased to 3.88 and 4.62 °brix with the application of 0.25 and 0.5% Zn as foliar spray, respectively (Table 4).

The interaction between Ca and B illustrated that the TSS content was significantly (*p* ≤ 0.05) higher (5.04 °brix) in control fruits. The least TSS (2.94 °brix) was recorded in fruits treated with 0.9% Ca + 0.5% B (Figure 9). The interaction between B and Zn was also found to be significant (*p* ≤ 0.05) and indicated that the highest TSS content (5.53 °brix) was recorded with a foliar application of 0.5% Zn + 0% B. By contrast, the minimum TSS content (3.28 °brix) of tomato fruits was recorded from the 0.5% B + 0% Zn treatment (Figure 9).

### 3.13. Percent Acidity (%)

A perusal of data revealed that the foliar application of Ca, B, and Zn did not significantly affect the percent acidity of tomato. The Ca × B, Ca × Zn, B × Zn, and Ca × B × Zn interactions were not significant for acidity (Table 4).

### 3.14. TSS to Acid Ratio

The foliar application of Ca, B, and Zn did not significantly affect the sugar acid ratio. The Ca × B interactions were significant, while the Ca × Zn, B × Zn and Ca × B × Zn interactions were not significant for TSS to acid ratio of tomato (Table 4).

A significant (*p* ≤ 0.05) interaction between Ca and B was observed for TSS to acid ratio and showed that the fruits of tomato plants treated with Ca at 0.6% in combination with 0% B showed the maximum TSS to acid ratio (23.47) as compared to the TSS to acid ratio of fruits harvested from plants sprayed with 0.9% Ca + 0.25% B (11.76) (Figure 10).

### 3.15. Ascorbic Acid Content (mg 100 g^−1^)

A perusal of the data showed that the foliar application of Ca and B did not significantly affect the mean ascorbic acid content of tomato fruit. However, the effect of Zn was significant on the ascorbic acid content of tomato. The Ca × B, Ca × Zn, B × Zn, and Ca × B × Zn interactions were not significant for ascorbic acid content (Table 5).

**Table 5:** Effect of calcium, boron, and zinc on ascorbic acid content, reducing and non-reducing of tomato.

Calcium Levels (%)	Ascorbic Acid Content (mg 100 g^−1^)	Reducing Sugars (%)	Non-Reducing Sugars (%)
0	10.19	2.80	1.13
0.3	10.56	2.81	1.19
0.6	12.83	2.73	1.23
0.9	12.07	2.75	1.39
LSD at α 0.05	NS	NS	NS
Boron (%)			
0	10.68	2.96	1.09
0.25	12.64	2.71	1.29
0.5	10.92	2.65	1.33
LSD at α 0.05	NS	NS	NS
Zinc (%)			
0	8.29 c	2.66 b	1.45 a
0.25	11.42 b	2.58 b	1.15 b
0.5	14.52 a	3.07 a	1.11 b
LSD at α 0.05	2.70	0.26	0.25
Interactions			
Ca × B	---	Figure 11	---
Level of Significance	NS	*	NS
Ca × Zn	---	Figure 11	Figure 12
Level of Significance	NS	**	**
B × Zn	---	---	---
Level of Significance	NS	NS	NS
Ca × B × Zn	---	---	---
Level of Significance	NS	NS	NS

Means followed by similar letter(s) in column do not differ significantly from one another. NS = non-significant and *, ** = Significant at 5, and 1% level of probability, respectively.

**Figure 11:**
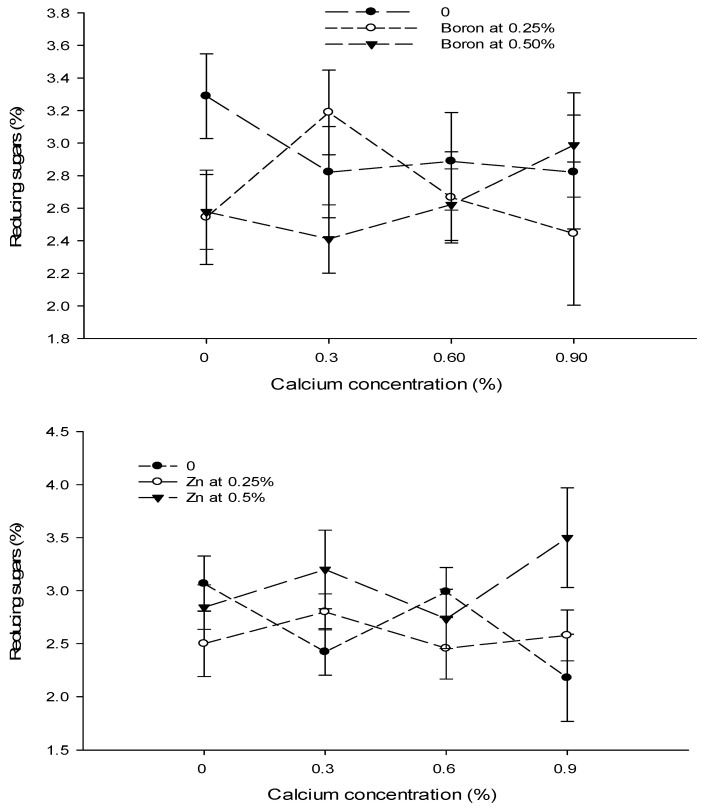
The interaction effects of Ca, boron, and Zn application on reducing sugars of tomato. The vertical bars represent standard error.

**Figure 12:**
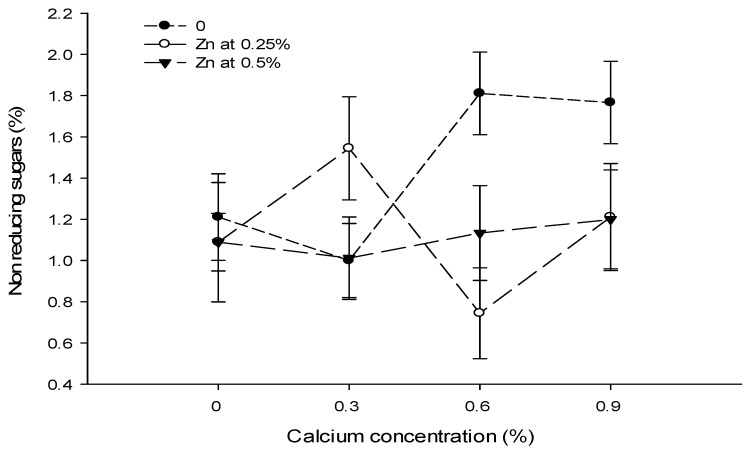
The interaction effects of Ca, boron, and Zn application on non-reducing sugars of tomato. The vertical bars represent standard error.

The ascorbic acid content of tomato increased with an increasing concentration of Zn from 0 to 0.5%. The highest ascorbic acid content (14.52 mg 100 g^−1^) was recorded at 0.5% Zn concentration followed by ascorbic acid of 11.42 mg 100 g^−1^ with a foliar application of 0.25% Zn. The least ascorbic acid content (8.29 mg 100 g^−1^) was recorded in control fruits (Table 5).

### 3.16. Reducing Sugars (%)

The data revealed that the foliar application of Ca and B did not significantly affect the reducing sugars of tomato fruit. However, the influence of Zn on the reducing sugars content of tomato fruit was significant. The Ca × B and Ca × Zn interactions were significant, while B × Zn and Ca × B × Zn interactions were not significant for reducing sugars (Table 5).

The reducing sugar content of tomato fruit enhanced with increasing the concentration of Zn from 0 to 0.5%. The reducing sugars of control fruits (2.66%) increased to 2.58% with the foliar application of 0.25% Zn solution. The reducing sugars increased to the maximum of 3.07% when the concentration of the foliar Zn solution was increased to 0.5% (Table 5).

The interaction between Ca and B was found to be significant (*p* ≤ 0.05). The reducing sugar content (3.28%) was the highest in control plants that decreased to 2.41% in tomato fruit harvested from plants treated with 0.3% Ca + 0.5% B (Figure 11). The interaction between Ca and Zn was found significant (*p* ≤ 0.05) for the reducing sugars of tomato. The lowest reducing sugars (2.18%) were found in fruits from plants that received a combination of 0.9% Ca + 0% Zn. By contrast, the maximum reducing sugars (3.50%) were recorded in the fruit of plants applied with 0.9% Ca + 0.5% Zn (Figure 11).

### 3.17. Non Reducing Sugars (%)

The data in Table 5 showed that the foliar application of Ca and B did not significantly affect non-reducing sugars. However, the influence of the foliar application of Zn significantly affected the non-reducing sugars of tomato fruit. The Ca × Zn interactions were significant, while Ca × B, B × Zn, and Ca × B × Zn interactions were not significant for non- reducing sugars (Table 5).

The foliar application of Zn had a concentration-dependent effect on the non-reducing sugar content of tomato fruit. The non-reducing sugars of tomato fruit decreased with an increasing concentration of Zn. The non-reducing sugars of control fruit (1.45%) declined significantly to 1.15% with a foliar application of 0.25% Zn solution. Increasing the Zn concentration of the foliar spray to 0.5% decreased the non-reducing sugar content to 1.11%, with the difference in 0.25 and 0.50% Zn foliar spray being non-significant (Table 5).

The interaction between Ca and Zn indicated that the non-reducing sugars of tomato were significantly (*p* ≤ 0.05) lower (0.74%) in fruits of plants treated with 0.6% Ca + 0.25% Zn, which increased to 1.81% where a 0.6% Ca + 0% Zn concentration was applied to the plants (Figure 12).

### 3.18. Correlation

**Figure 13:**
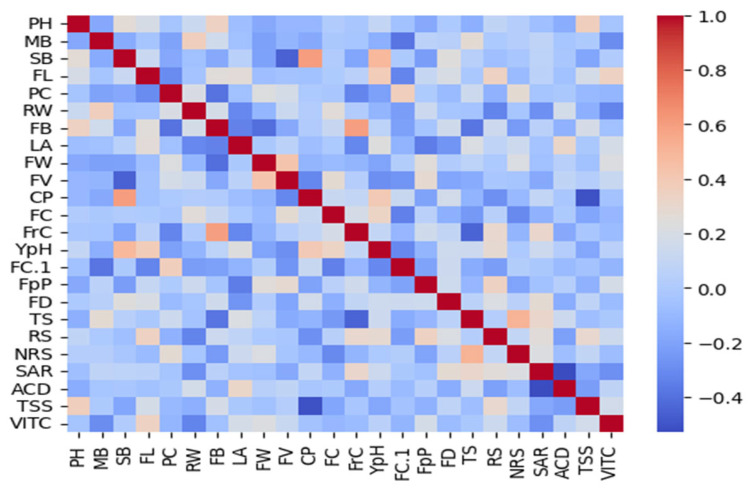
This heatmap visualizes the pairwise Pearson correlation coefficients, with red tones indicating positive correlations and blue tones indicating negative correlations. The intensity of the color corresponds to the strength of the correlation, with the scale ranging from −0.1 to 1.0. Diagonal cells show the autocorrelation of traits, illustrating the diverse interrelations and potential genetic or environmental influences on trait expression. CP: number of flower cluster plant^−1^, FC: number of flower cluster^−1^, FrC: number of fruit cluster^−1^, YpH: total yield, FB: BER incidence, FC1: fruit cracking, FD: flower drop, PH: calcium content, MB: boron content, SB: zinc content, TS: fruit firmness, TSS: total soluble solids, RS: reducing sugars, NRS: Non-reducing sugars, SAR: TSS to acid ratio, ACD: percent acidity, and VITC: ascorbic acid content.

Thermal correlation elucidates the pairwise relationships between the phenotypic traits assessed. Positive correlations were identified between closely related parameters, while notable negative correlations indicate potential antagonistic processes. The absence of correlation in other trait combinations reflects independent variations likely due to distinct underlying genetic or environmental influences. These insights provide a foundation for further investigation into the genetic architecture and physiological mechanisms governing trait development and interaction (Figure 13).

### 3.19. Principal Component Analysis

**Figure 14:**
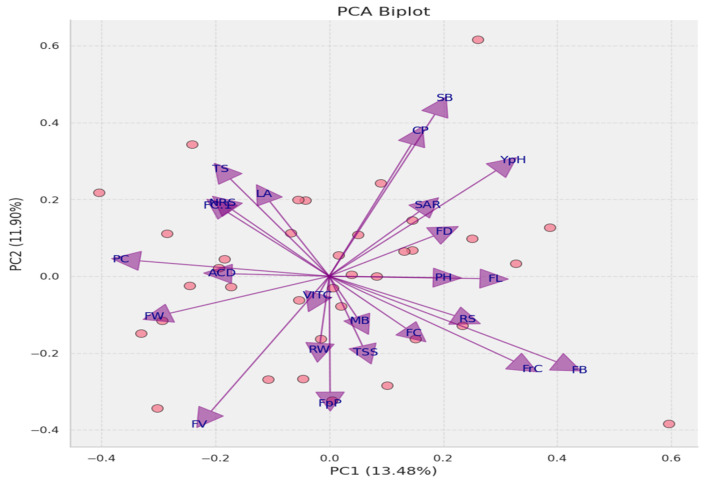
PCA biplot with varied trait contributions to principal components. The biplot displays the dispersion of individual plant samples and vectors representing key traits, with PC1 and PC2 explaining 13.48% and 11.90% of the variance, respectively. The trait vectors’ directionality and magnitude reflect their correlation and influence on the components, highlighting complex trait interplay within the dataset. CP: number of flower cluster plant^−1^, FC: number of flower cluster^−1^, FrC: number of fruit cluster^−1^, YpH: total yield, FB: BER incidence, FC1: fruit cracking, FD: flower drop, PH: calcium content, MB: boron content, SB: zinc content, TS: fruit firmness, TSS: total soluble solids, RS: reducing sugars, NRS: non-reducing sugars, SAR: TSS to acid ratio, ACD: percent acidity, and VITC: ascorbic acid content.

A PCA analysis explained the trait dynamics within the dataset, revealing that the first principal component (PC1) explained 13.48% of the total variance while the second principal component (PC2) accounted for 11.90%. The cumulative variance of 25.38% explained by PC1 and PC2 indicated that the dataset possesses a high-dimensional structure, reflecting the interaction of complex trait interplay. Biplot vectors demonstrate a wide range of correlations with the principal components, indicating varied trait contributions to the observed phenotypic variance. Traits such as FB, FC, and RS show strong positive correlations with PC1, while FV and FW are negatively correlated with PC1. These findings highlight the complex nature of the trait associations and underscore the potential for further dimensional exploration to understand the phenotypic diversity fully (Figure 14).

## 4. Discussion

The improvement in reproductive parameters, i.e., the number of flowers cluster^−1^, the number of fruits cluster^−1^, and the number of flower cluster plant^−1^ with calcium at 0.6 and 0.9% (Table 1), might be due to the fact that the calcium application increased the uptake of phosphorus needed to increase flower clusters [29], and enhancing the fruit set^−1^ in tomato plants [29], in addition to calcium inhibiting flower abscission, thus led to an increase in flower and fruit clusters^−1^ [39]. The foliar application of B also enhanced reproductive attributes (Table 1), which might be due to the fact that boron enhanced sugar levels of the stigma and helped in fruit set by promoting the germination of pollen and the growth of pollen tube [29,40,41]. Boron regulates carbohydrate metabolism [29] and increases the carbohydrate supply for flower formation and fruit set in tomato [38] as well as decreases flower abscission [38]. Thus, the boron application increased the flowers and fruit clusters^−1^ (Table 1). Zinc increased the number of flowers cluster^−1^ and fruit (Table 1) by increasing IAA synthesis [29,42] as well as carbohydrate translocation [29,31]. The foliar application of Zn also increased the photosynthate translocation to the fruit and decreased flowers and fruit abscission [29]. Thus, the Zn application may add to the number of flowers and fruits cluster^−1^, which confirmed the present results.

Both Ca and B promoted the flower and fruits cluster^−1^, indicating that Ca metabolism is enhanced by boron application [43,44]. Thus, the combination of Ca and B was more effective in increasing the flower and fruits cluster^−1^. Similarly, B and Zn promoted the translocation of carbohydrate from the site of formation to sinks that resulted in increased flower and fruit cluster^−1^ [39] which is evident from the present results as well (Table 1). Calcium and boron also decreased the abscission of flowers [38]. Thus, the combined application of Ca and B may have increased the number of flowers and fruits cluster^−1^ [38,40] by decreasing flower abscission [38]. The Zn has been found to be more effective in interactions with boron [42], and tomato plants treated with Zn and B resulted in high numbers of fruits plant^−1^ and higher yield than either nutrient applied alone [29,43] which is confirmed from the present results, as evident from Figure 1. Moreover, the application of Zn also increased the yield of tomato (Table 2), which might be due to the fact that zinc enhanced photosynthate transport to the fruits and played a role in the retention of flowers and fruits [13,29]. Moreover, the combined application of Ca and B also decreased flower abscission (Figure 3) and fruit retention [40] and resulted in higher yield as compared to the sole application of each nutrient [44]. Similarly, the combined application of Zn and B (Figure 3) also increased the yield more than either nutrient applied alone [29,31].

It is evident from Table 2 that calcium significantly suppressed the blossom end rot (BER) incidence in tomato. This might be due to the fact that BER is caused by a nutritional (NH_4_-N, K and Mg) imbalance that aggravates calcium deficiency, and thus reducing the Ca movement towards the fruits may enhance BER incidence [45]. In addition, high levels of both N and K are known to be involved in cell expansion, which may promote rapid fruit growth and lead to Ca deficiency [46]. It is suggested that localized calcium deficiency at the blossom end of the fruit initiates the BER disorder [12]. The blossom end rot disorder starts as cell wall damage and solute leakage occur [10,11,12] and minute cracks develop that expand further and open the route for wound pathogens to invade the fruit [8,9,10]. Thus, a preharvest calcium application reduces the blossom end rot incidence of tomato fruit [42], which is confirmed from the present results (Table 2). An optimum B supply, on the other hand, promotes the uptake of Ca, Mg, Na, and Zn and enhances the calcium metabolism in cell walls that decreases the BER incidence [41,47,48]. B enhances cell cross linking and promotes the cell wall structure, thus resulting in a decreased BER incidence [49]. Thus, a combined foliar application of Ca and B may have resulted in the maximum decline of BER incidence [50]. By contrast, a B deficiency may aggravate blossom end rot incidence by causing fruit cracking [51]. The application of Zn alone or in combination with boron also decreased the BER incidence (Figure 4). The foliar application of Zn raises the Zn content of the leaves and causes a decrease in blossom end rot [29,40], since Zn is involved in IAA biosynthesis [52]. A zinc application might increase the photosynthesis process [53] that results in an increasing leaf area [54], which is positively correlated with increased photosynthate production, e.g., sugar and chlorophyll content estimate the primary productivity and effect of environmental stresses [53]. Zinc provides a strong sink of photosynthates and their translocation to the fruits [53], hence more calcium to the fruit, thus resulting in less blossom end rot (Figure 4). Zinc stabilizes and protects bio membranes against oxidative stress and modulates plants’ antioxidant systems to reduce free radical damage effects [55], which may help to reduce physiological disorders (Figure 4). Several studies indicate that a foliar Ca application is an effective practice for preventing physiological disorders caused by a Ca deficiency in fruit species [5].

Fruit cracking is a physiological disorder that occurs due to calcium deficiency and fluctuations in temperature and water supply [13]. The fruit produced in such conditions develop a thinner monocarp that is weaker and more prone to cracking. Excessive irrigation or rainfall increases the soil moisture and water uptake that enhance fruit enlargement and the tomato skin with lower tensile strength resulting in the enlargement of minute cracks in the skin [11]. The cracks on the surface of the fruit serve as an entry point for pathogens [11,12,13,14]. The decrease in fruit cracking with a calcium application (Figure 5) is due to the role of calcium increasing cell turgidity by increasing the calcium bridges in the cell wall [56], thus increasing skin resistance to cracking [56]. The application of boron also decreases fruit cracking (Figure 5) by enhancing calcium metabolism and the cross linking of cell wall polymers [57]. Thus, boron and calcium have a synergistic effect in the cell wall [57], so it is possible to decrease cracking by improving cell wall resistance to cracking. The application of zinc also reduced fruit cracking [58]. Zinc applied also suppressed fruit cracking in tomato (Figure 5). This is due to the fact that zinc enhanced nutrient uptake and controlled water absorption as well as improved the IAA biosynthesis, which results in the decline of fruit cracking [52]. Zinc is also responsible for cell wall strengthening and decreases the abscission zone formation [59]. In addition, Zn performs so many regulatory roles in the development of plants by several enzymes’ activation, cell division and enlargement, and organic food biosynthesis, hence improving yield and quality by reducing the fruit cracking percentage [39,59]. The findings of this research are in accordance with [60,61]. They reported that a foliar application of Zn reduced cracking of the fruit in pomegranate.

The Ca deficiency causes sensitivity to several physiological disorders, such as fruit cracking as well as blossom end rot [8,10]. Thus, preharvest Ca is commonly used as foliar spray to increase the calcium content of fruit [20]. The increase in Ca content with increasing Ca concentration (Table 3) indicated that a foliar application was effective in increasing the Ca content of the fruit [20]. In this study, the maximum calcium content (Table 3) and minimum BER incidence was recorded with a 0.9% Ca application (Table 2). The findings of this study were in accordance with [8,10,20]. They reported that increased Ca content improved firmness and the lowest blossom end rot and fruit cracking. The Ca content of the fruit also increased with an increase in B concentration (Table 3), indicating that B promotes Ca metabolism and incorporation into the cell wall [20]. Thus, the application of B alone also increased calcium contents significantly. However, the influence of the Ca + B application (Figure 4 and Figure 5) was more than the sole application of Ca or B [26]. While the application of Ca alone had no effect on the fruit boron content (Table 3), it increased with the Ca × B interaction [62]. The foliar application of Zn alone decreased the calcium content of the fruit (Table 3).

The increasing levels of Zn significantly increased the Zn concentration in tomato fruit (Table 3). Almendros et al. [63] reported a high Zn concentration with soil or foliar applications of Zn. The zinc foliar application enhances Zn content of tomato (Table 3) and tomato seeds. Zinc uptake promotes the auxin levels in the plants and results in shoot growth, overall growth, nutrient uptake, and the control of disorders. The Zn foliar application increases Zn content, which promotes flower formation and fruit set that results in higher fruit yield [42] and juice quality [29,30,31]. The application of Zn enhances the photochemical reactions occurring in thylakoid membrane and electron transport through PSII and increases the rate of chlorophyll [29,30,31] and photosynthetic content [42]. A foliar supply of Zn increases the biosynthesis of chlorophyll and the carotenoid synthesis that are important for the proper performance of the photosynthetic process [29,30,31]. The foliar application of Zn increased gaseous exchange and maintained membrane integrity [29,30,31]. Ahmed et al. [64] also noted that an application of zinc increased the tomato Zn content, which had a positive effect on the vegetative and reproductive attributes of tomato. The increased level of boron decreased the Zn content of tomato fruit (Table 3). Asada et al. [62] also noted similar results and concluded that Zn content of tomato decreased with increased levels of boron in tomato.

Fruit firmness is an important quality parameter of tomato [65]. The pre-harvest calcium application enhances the calcium content of the fruit (Table 3) and results in increased fruit firmness (Table 4) [66]. The increased fruit firmness due to calcium application might be due to its accumulation in the cell wall, which promotes pectic polymers cross linking and cell wall strength [67]. The Ca and B treatment in combination resulted in greater firmness, indicating that boron enhanced the metabolism of calcium in the cell wall [68] by enhancing the cross-linked polymer network and making the cell wall firmer [69]. Zinc plays important regulatory and catalytic functions in plants, including sugar and starch synthesis and carbohydrate metabolism [70]; therefore, it may increase fruit TSS content and decrease the fruit firmness [71], which is evident from the present results (Table 4).

The TSS content is a rough estimate of sugar and other dissolved soluble content in fruit and vegetables [71]. The total soluble solids of tomato fruit are also decreased with a foliar calcium application (Table 4). The polysaccharides are degraded to simple sugars resulting in increased TSS during ripening [72]. The decrease in TSS with a calcium application might be the reason that it slows the ripening, and therefore, decreases the TSS. Moreover, the slower respiration retards the increase in TSS due to slower changes from carbohydrate to sugar [73].

The calcium application increases the fruit calcium content, which might be due to the fact that it influences changes associated with senescence, such as free sugars, anthocyanin content, organic acids, and fruit texture [74]. The boron [75] and zinc application [76] increased the total soluble solids of tomato fruit, probably by promoting carbohydrate metabolism [77]. The increase in TSS due to the Zn application could be due to its role in many regulatory and catalytic functions in plants, including sugar and starch synthesis and carbohydrate metabolism [70]. So, it may increase the TSS of the fruit [71], which is evident from the present results (Table 4). The increase in TSS content with boron and zinc may be attributed to the quick metabolic transformation of starch and pectin into soluble compounds and the rapid translocation of sugars from leaves to developing fruits [78].

Zn is involved in plant metabolism and has a key role in photosynthesis and related enzymes, resulting in increasing sugar and decreasing acidity [71]. The present results are confirmed from the results of Mishra et al. [79], who reported that ZnSO_4_ increased TSS in guava fruit.

Percent acidity and sugar acid ratio were not significantly affected by the Ca, B, and Zn foliar application. Haq [80] also reported similar results, who recorded a non-significant effect of Ca and B on quality attributes, especially the ascorbic acid content and sugar content of tomato. The foliar application of Zn alone increased the ascorbic acid and sugar content of tomato in the present study. Zinc in low concentration decreased the sugars of tomato but further increases in concentration increased the sugar content of tomato (Table 5).

High ascorbic acid content of tomato is a desirable characteristic of tomato [81] that is reported to increase by applying Zn [82]. The present results are in close conformity with Kumari [83], who reported that the application of zinc significantly improved the ascorbic acid content in tomato.

## 5. Conclusions

From the above results, it can be concluded that Ca at 0.6%, B at 0.25%, and Zn at 0.5% and their interactions significantly improved the vegetative, reproductive, and yield attributes of tomato, hence they are recommended for better quality tomato production. Moreover, 0.9% Ca, 0.5% B, and 0.25% Zn significantly reduced the physiological disorders and mineral attributes (fruit Ca content) of tomato and hence are recommended for tomato growers.

Future investigations need to be undertaken to check the status of Ca, Zn, and B in soil before conducting similar experiments. Furthermore, organic fertilizer and biological agents with these nutrients should be studied to overcome these and related disorders in tomatoes and other vegetables from an economical point of view.

The only limitation of this study was the erratic rainfall during the monsoon season, which may have led to an early drop and disease incidence in the tomato.

## Data Availability

The original contributions presented in this study are included in this article, and further inquiries can be directed to the corresponding authors.
